# Translation, Cultural Adaptation and Validation of a Korean Version of the Digital Professionalism Self‐Assessment Instrument for Nurses

**DOI:** 10.1155/jonm/7991455

**Published:** 2026-07-06

**Authors:** Suyeon Ban, Ja-Yeoung Lee, GyeongAe Seomun

**Affiliations:** ^1^ College of Nursing, Korea University, Seoul, South Korea, korea.ac.kr

**Keywords:** digital media, digital professionalism, e-professionalism, nurse, professionalism, psychological test, social media

## Abstract

**Background:**

Social media has become a crucial tool for interpersonal communication among nurses worldwide, including in South Korea. While offering various opportunities, such as education and networking, it also entails risks regarding professional ethics and boundaries. However, research measuring digital professionalism in the Korean context is inadequate. Thus, a customised, nurse‐specific instrument is required to support effective evaluation, educational initiatives and policy formulation.

**Aim:**

This study aimed to translate, culturally adapt and psychometrically validate the Korean version of the Digital Professionalism Self‐Assessment Instrument (K‐DP‐SAI) for nurses.

**Methods:**

This study followed established guidelines for translation and cross‐cultural adaptation, including forward and backward translation, synthesis, expert panel review and pilot testing. Exploratory and confirmatory factor analyses were conducted with 308 nurses each, and known‐group validity was also performed. Internal consistency was assessed using Cronbach’s alpha and McDonald’s omega and test–retest reliability was examined using intraclass correlation coefficients.

**Results:**

The K‐DP‐SAI comprises 14 items across three factors, including ‘upholding public trust through professional accountability’, ‘personal information protection’ and ‘maintenance of professional boundaries’. The scale demonstrated satisfactory content, convergent and discriminant validity with acceptable model fit indices (*x* ^2^/df = 2.603, GFI = 0.916, TLI = 0.918, CFI = 0.933, RMSEA = 0.072 and SRMR = 0.054) and also demonstrated sensitivity in discriminating between groups based on their perceived need for related education (*t* = 2.135, *p* < 0.05). Internal consistency (Cronbach’s alpha = 0.88 and McDonald’s omega = 0.88) and test–retest reliability (ICC = 0.68) met the established criteria.

**Conclusions and Implications for Nursing Management:**

The K‐DP‐SAI is a valid and reliable instrument for assessing nurses’ digital professionalism in South Korea. As an organisational safeguard, it enables managers to identify vulnerable groups and implement targeted, evidence‐based education beyond simple restriction. Ultimately, K‐DP‐SAI supports the nurses’ professional integrity in digital spaces by shifting management toward practical guidance.

## 1. Introduction

The healthcare environment is undergoing extensive changes in professional practice methods and communication structures, driven by advancements in digital technology and the rise of online platforms. Social media and online communication have emerged as important means for medical professionals to share information, foster collaboration and communicate with patients and the public [1]. Digital communication is reshaping how medical information is produced and disseminated, as well as how medical professionals interact with patients, further highlighting the need for professionals to reconsider their roles within the digital environment [2]. Crucially, nurses, as health professionals who interact closely with patients, are increasingly leveraging digital media for patient education, health information delivery and health promotion activities [3]. However, the expansion of the digital environment simultaneously has resulted in new ethical and professional challenges [4]. Specifically, in a digital environment typified by the rapid production and dissemination of information, the information presented by experts is likely to be accepted based on trust in the individual and the profession, rather than on the quality of the information itself. Consequently, low‐quality health information provided by experts can have a negative impact on the public’s health‐related decisions, posing an additional risk of contributing to the spread of incorrect information. Furthermore, the spatial attributes of online spaces, like anonymity, may undermine professional boundaries, leading to inappropriate online conduct or unethical postings [4, 5]. Within this environment, the protection of patient personal information and the maintenance of confidentiality are emerging as social issues [6].

Professionalism in the medical field is traditionally defined by professional values, attitudes and behaviours, which are core elements for sustaining patient and societal trust [7]. This traditional view is shaped primarily by direct interactions in clinical settings and inadequately accounts for professional behaviours manifested in public and informal online environments [4]. Consequently, a conceptual expansion is needed to understand new forms of professional behaviour and ethical responsibility in digital environments [2, 4, 8]. Digital professionalism refers to the attitudes and behaviours of medical professionals who maintain traditional professional values and behaviours in digital environments while utilising digital technology ethically and responsibly [4, 5]. The Korean Hospital Nurses Association has initiated practical research to establish professional guidelines for nurses’ online behaviour, responding to increased online engagement among nurses in Korea and social interest regarding their professional attitudes towards internet usage [9, 10]. Prior research has mainly focused on measuring technical competencies in digital literacy [11] and citizenship in digital spaces [12]. However, research on digital expertise in nursing, particularly concerning nurses’ clinical context, ethics and responsibilities in online use, remains insufficient. Furthermore, although a validated measurement tool is required to objectively assess and support nurses’ digital professionalism, to our knowledge, no academic research has addressed this topic.

The Digital Professionalism Self‐Assessment Instrument (DP‐SAI) evaluates attitudes and behaviours regarding social media use among healthcare professionals, particularly nurses. The DP‐SAI is based on eight professional guidelines, including those of the American Nurses Association (ANA) and the American Medical Association (AMA) and encompasses various domains, including anonymity, privacy, confidentiality, boundary setting, conflicts of interest, accountability, respect for peers and ethics. The DP‐SAI multidimensionally measures how professional values are perceived and performed in a digital environment [13]. It also functions as an indicator to assess the digital ethical awareness and professionalism of healthcare workers. Since the DP‐SAI was developed based on Western healthcare systems and professional cultural contexts, there may be differences in interpretation when applied to nursing environments in other cultures [14]. Importantly, Korea’s nursing organisations exhibit a relatively strong hierarchical structure; this organisational culture can influence nurses’ communication styles and behaviours. These cultural characteristics can influence responses to inappropriate social media use by colleagues and the maintenance of professional boundaries [15]. Furthermore, differences in Korea’s medical laws and personal information protection regulations, when compared to other countries, result in disparities in the ethical standards expected from professionals in the digital environment [16]. These cultural and institutional differences can lead to an application gap, where measurement tools developed abroad fail to adequately reflect the ethical conflicts and professional behaviours experienced in actual clinical practice [14]. Therefore, to apply internationally developed digital professionalism assessment tools to the Korean nursing environment, a systematic cross‐cultural adaptation process that considers cultural context is necessary [14, 17].

Accordingly, this study aimed to develop a Korean version of the DP‐SAI (K‐DP‐SAI) tailored for the Korean nursing context through a cross‐cultural adaptation process [13]. Furthermore, the objective is to verify the reliability and validity of the K‐DP‐SAI, to ensure its appropriateness as a standardised measurement tool intended to systematically evaluate Korean nurses’ digital professionalism. The K‐DP‐SAI is expected to provide important data for objectively measuring nurses’ digital professionalism and evidence‐based data for developing educational programmes and policies to strengthen nurses’ digital professional competencies in the future.

## 2. Methods

**FIGURE 1:**
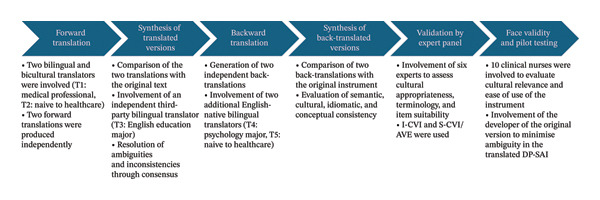
Process of translation and cross‐cultural adaptation of the K‐DP‐SAI.

The K‐DP‐SAI was developed in two distinct phases. Phase 1 utilised the six‐step guideline for translation and cross‐cultural adaptation from Sousa and Rojjanasrirat [14] and Cruchinho et al. [17] to ensure a rigorous adaptation and psychometric evaluation (Figure 1). The process is focused on the conceptual, item and semantic equivalence, addressing the cultural relevance within domains, the effective coverage of intended concepts by specific items and the preservation of original meaning and nuance, respectively [17]. Phase 2 involved the psychometric evaluation. This study was reported in accordance with STROBE checklist (Appendix 1).

### 2.1. Phase 1: Translation and Cross‐Cultural Adaptation

#### 2.1.1. Step 1: Forward Translation

The first author obtained permission via email from the original author to translate the DP‐SAI into Korean and verify its reliability and validity. Two native Korean translators independently translated the English version of the DP‐SAI, following specific guidelines and without prior knowledge of the original instrument, paper, or the planned retranslation. The first translator was a bilingual medical professional familiar with the terminology and ethics relevant to the instrument’s construct. The second translator, educated in both the United States and Korea, was culturally aware and fluent in both languages, but unfamiliar with healthcare field and instrument development.

#### 2.1.2. Step 2: Synthesis of the Translated Versions

The two translations were evaluated against the original source text by another Korean–American independent bilingual translator with expertise in education and English literature. This comparison aimed to uncover ambiguities or inconsistencies in meaning, vocabulary and sentence structure. Subsequently, the authors engaged in a discussion to refine the context’s relevance to the Korean nursing environment. Although structural differences between the two forward‐translated versions were insignificant, specific items were refined to better fit the Korean nursing context. For instance, Item 8 was reverted from ‘medical‐related queries’ to ‘healthcare queries’ to better align with the nursing scope, and ‘medical advice’ in Item 27 was adjusted to ‘health‐related advice’ to reflect professional boundaries. For Item 21, the scope of ‘entire healthcare profession’ was narrowed to ‘professional group’ to ensure the instrument remains versatile yet applicable to nursing without being overly broad. Ultimately, a unified translated version was developed.

#### 2.1.3. Step 3: Backward Translation

The integrated version of the translation process was back‐translated into the source language by two other independent translators, both native English speakers who were fluent in Korean. Neither had any prior knowledge of the original text of the instrument. The first professional translator had a degree in psychology and a conceptual understanding of psychological measurement and instruments. Having translated over 300 medical‐related papers, he was also fluent in medical terminology. The second translator was familiar with colloquial English, idiomatic expressions and emotive vocabulary but unfamiliar with the instrument’s medical terminology, theoretical underpinnings and the back‐translation process.

#### 2.1.4. Step 4: Synthesis of the Back‐Translated Versions

The translator (a linguistic expert) and the authors (nursing professionals and methodologists) involved in Step 2 discussed the semantic, cultural, idiomatic and conceptual consistency of the original, translated and back‐translated texts to create the pre‐final version in the target language, Korean. No significant differences in linguistic concepts and expressions between the two translations were observed. Given the constructs’ nature and their status as inherent professional expectations, the team made efforts to ensure that the items were not overly assertive.

#### 2.1.5. Step 5: Validation by the Expert Panel

The content validity was reviewed independently by six experts, including three professors specialising in nursing management, one in medical informatics and two managerial‐level nurses with over 15 years of clinical experience. It was assessed based on cultural appropriateness, appropriateness and ambiguity of terminology and items, and overall suitability. The experts provided descriptive feedback on these evaluations.

All 28 items were rated on a 4‐point Likert scale (1 = *strongly disagree*, 2 = *disagree*, 3 = *agree* and 4 = *strongly agree*). The content validity index (CVI) was assessed at the item (I‐CVI) and scale (S‐CVI) levels. CVI scores of 1 and 2 were recoded as disagree (0), whereas scores of 3 and 4 were recoded as agree (1). The I‐CVI was calculated by dividing the number of experts who agreed by the total number of experts. The S‐CVI/average (S‐CVI/AVE) was calculated by summing all I‐CVI scores and dividing them by the total number of items. For a group of 6–10 experts, minimum thresholds of I‐CVI and S‐CVI/AVE should be 0.78 and 0.90, respectively [18]. In this study, the I‐CVI ranged from 0.83 to 1 for each item, whereas the S‐CVI/AVE was 0.97. No items were eliminated.

Nevertheless, some were modified based on expert opinions. Among the original items, the terms ‘entertain’ and ‘attention’ in Item 8 (‘I do not entertain my patient’s queries about healthcare if they contact me through my private/personal social media profile’) and Item 24 (‘If I see unprofessional content posted by my colleague, I feel responsible to bring it to the attention of that person’) may convey unintendedly strong connotations of dismissal, caution, or criticism in the Korean cultural context. Therefore, they were modified to convey a more appropriate gentle warning while respecting the subject and colleagues. The phrases were changed to ‘Respond saying that an answer cannot be given’ (Item 8) and ‘Notify them that attention is required’ (Item 24).

In Item 13, which addresses patients’ identifiability in private online forums, the term ‘online forum’ was considered inappropriate for the Korean context, as it is rarely used. To better reflect an open discussion space and align with the community concept, it was revised to ‘private online community’.

Item 20, which refers to compliance with social media guidelines for professional use, was revised due to the absence of such guidelines for nurses in Korea. It was revised to glean information about perceptions of complying with such guidelines to ensure cultural appropriateness.

Article 34 of the Korean Medical Service Act defines medical advice as telemedicine, a practice restricted to doctors, dentists and oriental medicine doctors [16]. In Item 27, the phrase ‘giving health‐related advice during online interactions’ was revised to ‘provision of health information’ to avoid being misinterpreted as medical advice, which could conflict with the legal scope of nursing.

#### 2.1.6. Step 6: Face Validity and Pilot Testing

A pilot study and face validity assessment were conducted using the preliminary version of the K‐DP‐SAI developed in Step 5. The recommended sample size for face validity is 10–40 [14]. A preliminary questionnaire was administered to 10 clinical nurses who use social media at least once a day. They were asked to evaluate whether (1) any sentences were difficult to understand, (2) the context and terminology were awkward or needed revision and (3) vocabulary and items were culturally inappropriate. Their responses were rated on a discreteness scale (clear or unclear). An ‘unclear’ score of less than 20% was considered acceptable [14]. Nurses were also asked to provide descriptive feedback on any parts they found inconvenient or incomprehensible, based on which some items were revised. During the item revision process, the authors discussed areas of disagreement, directions for revision and unclear meanings in the Korean context with the original corresponding author as below.

For example, in item 6, although the original author intended to convey that social media ‘privacy settings are imperfect’, reflecting their inherent limitations, the participants interpreted the item as implying a causal relationship between inadequate privacy settings and the widespread disclosure of content. Therefore, it was rephrased as ‘because privacy settings are not perfect’ to convey the intended meaning clearly.

Across the items, the use of the term ‘professional social media’ in the DP‐SAI was considered ambiguous in the Korean context. The original author stated that it referred to social media used for work or business purposes. However, in Korea, nurses are legally prohibited from establishing or operating their clinic, and the use of professional social media is largely limited to specific messaging applications. This creates a contextual conflict between the Korean setting and the original author’s intended meaning. Therefore, the authors of this study agreed to expand the definition of ‘professional social media’ to encompass all online platforms used by nurses based on their professional identity, including social media for work purposes. To facilitate understanding, the meaning of ‘professional social media’ was explained in the questionnaire.

Additionally, Item 20, which had been revised based on expert feedback during the content validity from ‘complying with social media guidelines’ to ‘being aware of the need to adhere to social media use’—considering the limitations of such guidelines for Korean nurses—remained an item of concern during the face validity evaluation. After consultation with the original author, the item was revised again to ‘using professional social media platforms in accordance with professional guidelines’.

Accordingly, the final version was established, and the pilot test subjects were excluded from Phase 2 [19].

### 2.2. Phase 2: Validation of the K‐DP‐SAI

#### 2.2.1. Study Sample

To verify the validity and reliability of the scale, 616 registered nurses were recruited through an online survey by EMBRAIN, a panel provider, from 3 to 10 July 2025. Participants were required to work in clinical settings and use at least one social media platform (such as Facebook, Instagram, YouTube, KakaoTalk, or blogs) at least once a day, while those not involved in direct patient care were excluded. Based on the recommendation of having at least 10 participants per item, a minimum sample size of 280 was required for each of the exploratory factor analysis (EFA) and confirmatory factor analysis (CFA) [20–22]. To accommodate a 10% dropout rate, a total of 616 participants were recruited. The participants were divided into two independent groups, comprising Group A (*n* = 308) for EFA and Group B (*n* = 308) for CFA, using stratified randomisation based on sex and age via random numbers generated in Microsoft Excel. From Group B, the sample size for the test–retest reliability assessment was determined considering an expected dropout rate, 80% power, *α* = 0.05 and an intraclass correlation coefficient (ICC) target of 0.7 [23]. Consequently, 11 participants were recruited for this assessment. The data were analysed using SPSS Version 29.0 and AMOS 29.0.

#### 2.2.2. Participant Characteristics

General characteristics of the nurses who participated in the study were analysed using indicators such as mean ± standard deviation (SD), frequency and percentage. The analysed detailed information included nurses’ sex, age, education level, clinical experience (months), work experience (months), hospital type, work unit, duration of social media use (months), daily social media use (hours), number of social media platforms and purpose of social media use.

#### 2.2.3. Item Analysis

To assess item bias and normality of the data used in EFA and CFA, an item analysis was conducted by examining the mean, SD, skewness, kurtosis and item–total correlation for each item. Normality was assessed using criteria such as skewness less than 2 and kurtosis less than 4 to avoid overfitting [24]. Items with item–total correlations less than 0.4 or greater than 0.8 were removed [25].

#### 2.2.4. Construct Validity

The structural validity of the K‐DP‐SAI was verified using EFA and CFA. EFA was conducted using principal component analysis (PCA) with varimax rotation. Prior to the analysis, the adequacy of the factor analysis scale was assessed using the Kaiser–Meyer–Olkin (KMO) test (> 0.80) and Bartlett’s test of sphericity (*p* < 0.05) [26]. Factor loadings and communality were examined to assess how well each item reflected the characteristics of construct validity. Factors were identified when eigenvalues were 1 or greater and when factor loadings and communality were 0.40 or greater [27, 28]. If cross‐loading exceeded the 0.40 threshold, items showing loading differences of less than 0.20 between the main and subfactors were removed [29].

CFA was performed using the maximum likelihood estimation method, and model fit was evaluated with chi‐square tests (*χ* ^2^/df), goodness‐of‐fit index (GFI), comparative fit index (CFI), Tucker–Lewis index (TLI), root mean square error of approximation (RMSEA) and standardised root mean square residual (SRMR). A *χ* ^2^/df value of less than 3 indicates good model fit [30]; GFI, CFI and TLI values ≥ 0.90 are considered appropriate, and values ≥ 0.80 are deemed acceptable [31, 32]. RMSEA and SRMR below 0.08 are generally regarded as indicating a reasonable fit [30, 33]. Convergent validity was assessed using standardised regression weights (SRW), average variance extracted (AVE) and composite reliability (CR) and considered adequate when SRW and AVE were ≥ 0.50 with statistically significant SRW values [34, 35], and the CR was ≥ 0.70 [36]. Discriminant validity was considered adequate when AVE > Ф^2^ [35, 37, 38].

In the absence of an established ‘gold standard’ for this relatively new concept, criterion validity was unavailable. Consequently, we conducted known‐groups validity testing, using the perceived need for education on social media ethics and professionalism as a grouping variable [25]. This was based on the reasoning that a recognised need for such education may reflect a higher sensitivity to the ethical dimensions and the complexity of professional interactions in digital environments [5]. An independent *t*‐test was performed to compare the mean scores between the two groups.

#### 2.2.5. Reliability Analysis

To assess internal consistency reliability, Cronbach’s *α* and McDonald’s *ω* were calculated for each factor and the overall scale, with values ≥ 0.70 considered acceptable [39]. Test–retest reliability was evaluated using the ICC, which measures the agreement between repeated measurements [40]. To minimise memory effects, retests were conducted at intervals of 2–4 weeks [21]. An ICC of 0.60–0.74 indicates good agreement [40].

### 2.3. Ethical Considerations

This study was conducted in accordance with the Declaration of Helsinki and received approval from the Korea University Institutional Review Board on 13 January 2025 (KUIRB‐2025‐0017‐01). All the participants were informed of the study objectives and procedures, assured of data confidentiality and voluntarily agreed to participate. Written informed consent was obtained for content validity and the preliminary assessment. For the online survey, participants provided their informed consent by clicking a checkbox to indicate agreement after reading the explanatory statement. All instructions and consent procedures were pre‐approved by the Korea University Institutional Review Board, ensuring compliance with ethical standards for research involving human participants.

## 3. Results

### 3.1. Participant Characteristics

This study analysed data from 616 clinically employed nurses who used social media. Participants’ demographic information is presented in Table 1. Participants were predominantly female, and their ages ranged from 23 to 66 years. Most had a college degree or higher educational qualification and worked in general hospitals.

**TABLE 1:** Baseline participant characteristics.

		Pilot (*N* = 10)	EFA (*N* = 308)	CFA (*N* = 308)
		*n* (%)/mean ± SD	*n* (%)/mean ± SD	*n* (%)/mean ± SD
Gender	Male	1 (10)	24 (7.8)	24 (7.8)
	Female	9 (90)	284 (92.2)	284 (92.2)

Age		34.30 ± 7.59	36.59 ± 8.79	36.48 ± 8.80

Degree	Associated	None	59 (19.2)	49 (15.9)
	Bachelor	6 (60)	228 (74)	227 (73.7)
	Master	3 (30)	19 (6.2)	32 (10.4)
	Doctoral	1 (10)	2 (0.6)	—

Clinical experience (months)		136.7 ± 104.83	131.93 ± 92.66	127.97 ± 90.94

Work experience (months)		94.6 ± 96.11	60.02 ± 55.10	54.53 ± 57.09

Hospital type	Private	None	85 (27.6)	98 (31.8)
	General	1 (10)	123 (39.9)	128 (41.6)
	Tertiary	9 (90)	100 (32.5)	82 (26.6)

Work unit	Ward	3 (30)	157 (51)	154 (50)
	ICU	6 (60)	33 (10.7)	22 (7.1)
	ER	None	13 (4.2)	16 (5.2)
	Outpatient	None	56 (18.2)	75 (24.4)
	OR/ANE	1 (10)	20 (6.5)	25 (8.1)
	PA	None	29 (9.4)	16 (5.2)

Duration of social media use (months)		141.5 ± 75.00	124.32 ± 65.50	131.70 ± 70.10

Daily social media use (hours)	4 (40)	70 (22.7)	62 (20.1)	20.1
	5 (50)	172 (55.8)	185 (60.1)	60.1
	1 (10)	66 (21.4)	61 (19.8)	19.8

Number of social media platforms		Not measured	2.5 ± 1.16	2.36 ± 1.03

Purpose of social media use^∗^	Personal commination	9 (90)	246 (79.9)	241 (78.2)
	Work‐related communication	2 (20)	104 (33.8)	92 (29.9)
	Information search	8 (80)	250 (81.2)	254 (82.5)
	Professional networking	None	36 (11.7)	33 (10.7)
	Leisure	None	5 (1.6)	3 (1)

*Note: N* = total number of sample; *n* = portion of total sample.

Abbreviations: ANE, anaesthesiology; ER, emergency room; ICU, intensive care unit; OR, operation room; PA, physician assistant; SD, standard deviation.

^∗^Multiple responses are allowed.

The average clinical experience was 128 months for Group A and 132 months for Group B. General nurses accounted for over 70% of both groups. More than 50% of the nurses worked in wards, and other positions included intensive care units, emergency rooms, outpatient clinics, surgery, anaesthesia and specialised nursing. Average departmental experience was 60 months for Group A and 55 months for Group B.

The average duration of social media use was 124 months for Group A and 132 months for Group B. More than 50% of both groups used social media for 1–3 h per day. Group A used 2.5 social media platforms, while Group B used 2.36, on average. The most common reasons for using social media were to search for information, share content and remain in contact with acquaintances and friends. Additional information on nurses’ perception of social media use is provided in Appendix 2.

### 3.2. Item Analysis

Item analysis of the data for EFA and CFA was conducted using skewness, kurtosis, adjusted item–total correlations and the means and SDs of each item (Table 2). Item means ranged from 2.1 to 4.69, and SDs ranged from 0.63 to 1.24. Items 11 and 25 were excluded because their absolute skewness exceeded 2. The absolute skewness values ranged from 0.32 to 1.80, while the kurtosis values ranged from 0.01 to 3.95; the normality criterion was met. Adjusted item–total correlations were 0.40 or higher for all items, except Items 1 and 5, which were subsequently removed for failing to meet this criterion.

**TABLE 2:** Item analysis.

Item	Original factor	Mean	SD	Skewness	Kurtosis	Item–total correlation
q1	Self‐anonymity	2.10	1.13	0.71	−0.50	−0.06
q2	Self‐anonymity	4.22	0.94	−1.30	1.45	0.443
q3	Self‐anonymity	4.00	0.86	−0.78	0.49	0.499
q4	Privacy settings	4.02	0.90	−0.84	0.57	0.482
q5	Privacy settings	3.47	1.14	−0.32	−0.72	0.352
q6	Privacy settings	4.15	0.80	−0.70	−0.01	0.594
q7	Privacy settings	4.14	0.81	−0.73	0.27	0.602
q8	Maintaining boundaries	3.87	1.04	−0.73	0.03	0.436
q9	Maintaining boundaries	4.48	0.88	−1.80	2.72	0.580
q10	Maintaining boundaries	4.44	0.85	−1.49	1.75	0.584
q11	Maintaining confidentiality	4.67	0.69	−2.38	6.03	0.712
q12	Maintaining confidentiality	4.49	0.79	−1.68	2.74	0.680
q13	Maintaining confidentiality	4.36	0.84	−1.36	1.53	0.695
q14	Conflict of interest	4.30	0.87	−1.24	1.49	0.648
q15	Conflict of interest	4.37	0.87	−1.49	2.03	0.706
q16	Conflict of interest	4.54	0.78	−1.70	2.35	0.615
q17	Accountability	4.50	0.76	−1.52	2.00	0.705
q18	Accountability	4.50	0.76	−1.44	1.29	0.730
q19	Accountability	4.56	0.70	−1.61	2.55	0.747
q20	Accountability	3.77	1.24	−0.73	−0.39	0.451
q21	Accountability	4.18	0.86	−0.94	0.75	0.615
q22	Respect for colleague	4.61	0.68	−1.72	2.38	0.703
q23	Respect for colleague	4.44	0.76	−1.33	1.58	0.704
q24	Respect for colleague	4.00	1.04	−0.80	−0.23	0.525
q25	Ethics	4.69	0.63	−2.11	3.95	0.739
q26	Ethics	4.55	0.70	−1.42	1.20	0.724
q27	Ethics	4.49	0.77	−1.76	3.48	0.769
q28	Ethics	4.52	0.69	−1.28	0.95	0.753

Abbreviation: SD, standard deviation.

### 3.3. Construct Validity

#### 3.3.1. EFA

Following the item analysis, EFA was performed using PCA on Group A (*n* = 308). The KMO value was 0.901, and Bartlett’s test of sphericity was statistically significant (*χ* ^2^ = 1946.919, df = 91, *p* < 0.001), indicating that the data were suitable for factor analysis. The PCA results revealed that the K‐DP‐SAI was extracted into 3 factors and 14 items. The total explanatory power was 62.26% (Factor 1: 29.73%, Factor 2: 18.78% and Factor 3: 13.75%). The EFA results are presented in Table 3. All item loadings were 0.50 or higher, and communalities were 0.40 or higher. Regarding cross‐loadings, most items remained below the 0.35 threshold, except for Item 10, which showed a secondary loading of 0.36 but a primary loading of 0.795 on Factor 3. Following the criterion that no cross‐loading is considered to occur when the difference in factor loadings is greater than 0.1, we judged these to be distinct factors [27]. We named each factor to encompass all items while remaining within the scope of the original measurement. Accordingly, Factors 1 to 3 were labelled ‘upholding public trust through professional accountability’, ‘personal information protection’ and ‘maintenance of professional boundaries’, respectively.

**TABLE 3:** Exploratory factor analysis (*N* = 308).

Factor	Question no.	Factor loading	Communality	Eigenvalue	Variance (%)	Cumulative variance (%)
1	28	0.809	0.707	4.161	29.725	29.725
	27	0.801	0.723			
	18	0.774	0.651			
	22	0.772	0.662			
	13	0.731	0.578			
	15	0.681	0.556			
	21	0.530	0.400			

2	3	0.773	0.633	2.630	18.784	48.509
	7	0.750	0.676			
	6	0.729	0.662			
	4	0.718	0.536			

3	10	0.795	0.764	1.925	13.753	62.261
	9	0.780	0.711			
	8	0.575	0.458			

*Note:* Factor 1: upholding public trust through professional accountability. Factor 2: personal information protection. Factor 3: maintenance of professional boundaries.

#### 3.3.2. CFA

**FIGURE 2:**
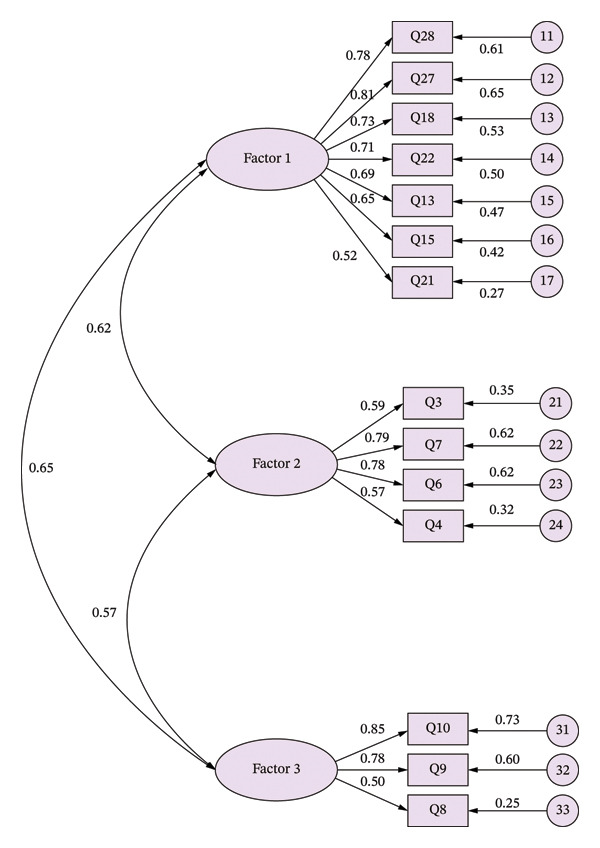
Structure model of the K‐DP‐SAI. *Note:* Factor 1: upholding public trust through professional accountability. Factor 2: personal information protection. Factor 3: maintenance of professional boundaries.

CFA was performed using the data from Group B (*n* = 308). The model fit indices were *x* ^2^/df = 2.603, GFI = 0.916, TLI = 0.918, CFI = 0.933, RMSEA = 0.072 and SRMR = 0.054, all within recommended limits. SRW ranged from 0.515 to 0.854, exceeding the 0.5 threshold for all items except for Item 8, at 0.496. All SRW values were statistically significant (*p* < 0.001). The AVE and CR values ranged from 0.546 to 0.640 and 0.775 to 0.925, respectively, establishing convergent validity (Table 4, Figure 2). The squares of all possible factor correlation coefficients were lower than the AVE values of the factors (AVE > Ф^2^), indicating sufficient discriminant validity (Table 5). Although its SRW was 0.496, Item 8 was retained based on its statistical significance, satisfied construct validity through AVE and CR, and its essential role in measuring active boundary management as a critical aspect for Factor 3 distinct from the pre‐emptive nature of the other items within the same factor [36]. In terms of known‐groups validity, the group perceiving a need for education demonstrated a statistically significant higher overall K‐DP‐SAI mean score (*t* = 2.135, *p* < 0.05). A significant difference was also observed in Factor 1, ‘upholding public trust through professional accountability’ (*t* = 2.195, *p* < 0.05), validating the scale’s ability to differentiate levels of digital professionalism between groups (Table 6).

**TABLE 4:** Confirmatory factor analysis and reliability test (*N* = 308).

Confirmatory factor analysis						Internal consistency reliability	
Factor	Question no.	SRW	Error variance	AVE	CR	Cronbach’s α	McDonald’s *ω*
1	28	0.780	0.173	0.640	0.925	0.858	0.858
	27	0.809	0.145				
	18	0.730	0.216				
	22	0.710	0.183				
	13	0.686	0.341				
	15	0.649	0.381				
	21	0.515	0.504				

2	3	0.594	0.482	0.560	0.832	0.776	0.774
	7	0.785	0.254				
	6	0.784	0.256				
	4	0.566	0.507				

3	10	0.854	0.170	0.546	0.775	0.727	0.737
	9	0.776	0.323				
	8	0.496	0.819				

Total						0.883	0.880

*Note:* Factor 1: upholding public trust through professional accountability. Factor 2: personal information protection. Factor 3: maintenance of professional boundaries.

Abbreviations: AVE, average variance extracted; CR, composite reliability; SRW, standardised regression weights.

**TABLE 5:** Discriminant validity (*N* = 308).

	Factor 1	Factor 2	Factor 3
Factor 1	**0.640**		
Factor 2	0.381	**0.560**	
Factor 3	0.424	0.331	**0.546**

*Note:* Factor 1: upholding public trust through professional accountability. Factor 2: personal information protection. Factor 3: maintenance of professional boundaries. Bold values on the diagonal represent the average variance extracted (AVE). Below‐diagonal values indicate squared interfactor correlations.

**TABLE 6:** Known‐group validity (*N* = 308).

Factor	Those perceiving a need for education (*n* = 272)		Those perceiving no need for education (*n* = 36)		*t*	*p*
	Mean	SD	Mean	SD		
1	31.78	3.6	30.08	4.44	2.195	< 0.5
2	16.39	2.54	15.86	3	1.155	0.249
3	13.14	2.07	12.69	3.14	1.132	0.258
Total K‐DP‐SAI	61.31	6.8	58.64	8.76	2.135	< 0.5

*Note:* Factor 1: upholding public trust through professional accountability. Factor 2: personal information protection. Factor 3: maintenance of professional boundaries.

Abbreviation: SD, standard deviation.

### 3.4. Reliability

Internal consistency was assessed using Cronbach’s α and McDonald’s *ω*, while test–retest reliability was assessed using ICC. Cronbach’s α and McDonald’s *ω* were 0.883 (0.727–0.858) and 0.880 (0.737–0.858), respectively (Table 4), and ICC was 0.679.

## 4. Discussion

Nurses’ increasing use of social media has highlighted concerns and expectations regarding its professional use. In response to the growing relevance of social media, this study translated, culturally adapted and validated the K‐DP‐SAI for use among Korean nurses. The findings indicate that digital professionalism in the South Korean nursing context comprises three dimensions: upholding public trust through professional accountability (7 items), personal information protection (4 items) and maintenance of professional boundaries (3 items). Although the original DP‐SAI comprised 28 items across eight domains, the Korean version demonstrated a more consolidated structure. Nevertheless, satisfactory psychometric findings support the validity of this structure in the South Korean nursing context.

Regarding the parsimonious construct of the K‐DP‐SAI, the primary finding was the emergence of Factor 1 as the most prominent dimension, defined as ‘upholding public trust through professional accountability’. This factor’s statistical dominance suggests Korean nurses perceive professional accountability, not as a collection of isolated tasks, but as the core pillar of their digital presence. This structural shift would reflect a seamless extension of integrated clinical ethics into the digital sphere. In clinical practice, nursing ethics is viewed as a holistic, inseparable responsibility rather than a fragmented set of rules; our findings suggest that South Korean nurses apply this same unified lens online [41]. They view the digital landscape as a professional arena necessitating ongoing, deliberate management. However, the nature of digital platforms, including social media, further intensifies the necessity for such integration. While the core ethical principles remain constant, the potential impact of an ethical lapse is extremely pervasive and irreversible [42]. For instance, the online disinhibition effect can lead to unintended ethical lapses [43], while the inherent halo effect associated with the nursing profession could disproportionately amplify the impact on patient safety and public trust [44]. Although direct cross‐cultural comparisons are currently limited due to the lack of international validation studies, acknowledging these amplified internal and external risks, South Korean nurses seem to exercise heightened caution, unifying respect (Items 22 and 28), patient safety (Items 22 and 27) and confidentiality (Item 13) into a single accountability dimension. This proactive stance demonstrates that they perceive individual vigilance as a direct and essential link to the preservation of the profession’s collective integrity. Ultimately, Factor 1 represents a unified ethical front designed to safeguard the trust of the nursing profession across both clinical and digital environments.

While Factor 1 denotes the internalisation of professional identity, Factors 2 and 3 reflect a greater awareness of the digital landscape’s technical and social vulnerabilities. Factor 2, initially focused on ‘privacy settings’, has evolved to encompass social media’s inherent traits—notably its pervasive publicity (Items 3 and 6) and content persistence (Item 7)—alongside proactive strategies for protecting personal information (Item 4). The integration of Item 3 further suggests that South Korean nurses perceive the limitations of anonymity as an extension of social media’s public character rather than a separate construct. This factor structure’s consistency with the original instrument indicates that these elements of digital professionalism form a universal construct, transcending cultural boundaries [13], and are consistent with South Korean research that identifies account management as a critical component of digital professionalism [45]. Such cross‐cultural similarity is rooted in shared knowledge that online information can be inadvertently reframed or disseminated beyond the user’s intent [4, 6]. For nursing professionals, the absence of digital boundaries poses an immediate professional challenge, as their personal conduct is constantly under public scrutiny [5, 6]. In this regard, Factor 2 functions as a proactive strategic tool to establish clear digital boundaries, allowing nurses to manage the blurring of private and professional lives, thereby sustaining their professional integrity and credibility [46, 47]. Ultimately, this safeguard would enable nurses to interact in the digital sphere with reduced personal or collective liability risk [5].

Factor 3, addressing professional boundary maintenance, preserves the original factor structure, akin to Factor 2. It includes both pre‐emptive measures to limit private relationship formation (Items 9 and 10) and reactive adjustments to regulate existing digital interactions (Item 8). This reflects the increasingly fluid nature of the digital landscape, where traditional clinical boundaries between nurses and patients are progressively eroded [48]. In the pre‐digital era, professional interactions were largely confined to clinical settings, whereas social media and digital visibility has fostered an environment of mutual observation where patients and nurses can engage outside the bedside practices [6, 49]. This increased transparency can obscure the professional boundaries designed to protect patient vulnerability. According to research, social media interactions may shift professional relationships in informal directions. These interactions may distort therapeutic boundaries and foster inappropriate dependency, which can confuse professional responsibilities [4, 5]. Consequently, while maintaining the original factor structure, the convergence of these items into Factor 3 indicates that South Korean nurses believe that digital boundaries are a core and universal element of their digital professionalism, and to uphold professional integrity within this sphere requires regulating these borders.

As noted in previous studies, translated terminology via a rigorous forward and backward translation process often undergoes conceptual shifts when applied to different cultural landscapes, leading to a natural refinement of the instrument’s content to ensure contextual relevance [50]. Several contextual factors may explain these changes. First, the difference in the primary target population significantly influenced the results. The original DP‐SAI was validated for a broad range of healthcare professionals and may not fully capture the nuanced experiences of nurses, who often approach social media with greater caution than their medical counterparts. For instance, while a key tenet of digital professionalism involves proactively managing one’s online identity, which is a practice increasingly promoted in the medical profession to enhance visibility and public trust, the nursing profession continues to perceive social media through a lens of risk management, particularly regarding patient confidentiality and professional conduct [3]. This cautious stance may prevent nurses from publicly disclosing their name and occupation (Items 1 and 2) or sharing personal opinions on health‐related matters online (Item 21). Consequently, distinguishing personal from institutional opinions may feel conceptually distant or culturally unfamiliar in the current nursing context. Second, the lack of institutional infrastructure contributed to interpretive ambiguity. In the absence of specific social media guidelines or structured e‐professionalism education for nurses in South Korea, items referring to ‘adhering to guidelines’ (Item 30) remained vague despite multiple rounds of cultural adaptation [51]. Furthermore, the responsibility to address a colleague’s unprofessional behaviour (Item 24) posed a significant cultural mismatch. In a hierarchical culture rooted in seniority, the act of ‘bringing attention’ to a peer’s conduct may be perceived as socially inappropriate rather than an act of professional respect [15].

Due to the lack of a gold standard measurement or equivalent validated instruments in South Korea, analysing criterion validity or examining construct validity against another measure was not possible in this study. Instead, known‐group validity was conducted by comparing groups according to their perceived need for education in social media ethics. The group that perceived a need for training demonstrated statistically significantly higher overall K‐DP‐SAI scores and Factor 1 scores. These results validate the K‐DP‐SAI’s sensitivity in discriminating between groups with differing levels of digital professionalism. Additionally, these findings suggest that nurses who acknowledge the need for training tend to have a heightened awareness of digital complexities. Conversely, the lower scores among those perceiving no need for training may indicate a blind spot where practitioners underestimate ethical risks or overstate their own competence. Therefore, nursing leadership must investigate whether this indifference towards digital conduct stems from a gap in actual digital proficiency, where individuals overestimate their skills, or from previous educational experiences focussing largely on restricting online use rather than promoting practical and professional engagement, or from other underlying reasons.

### 4.1. Implication for Nursing Management

The validated K‐DP‐SAI is not intended as a tool for surveillance or restriction but as an instrument that facilitates a supportive, professional environment in which digital technology reinforces nurses’ professional pride and ethical integrity. Effective management begins with identifying specific needs rather than imposing uniform regulations. The K‐DP‐SAI provides a means to identify vulnerable areas, such as patient privacy and professional boundaries. This allows managers to recognise groups at higher risk and prioritise resources for targeted education, ensuring that organisational interventions are supportive and evidence‐based.

Furthermore, nurses, lacking structured education and clear institutional guidelines, are considerably influenced by online peer networks. Without a formal framework, they may be exposed to unprofessional content from colleagues, which may gain social approval through ‘likes’. This ambiguity fosters a hidden curriculum, causing nurses to misinterpret such behaviours as accepted professional standards. The K‐DP‐SAI can function as an organisational safeguard by revealing these gaps, enabling leaders and preceptors to reinforce their roles as digital role models who guide staff away from inappropriate online trends.

Ultimately, online engagement is an inevitable and irreversible trend in the modern era. Therefore, the cultivation of digital professionalism could help nurses benefit from these digital spaces while maintaining their professional standing. In this regard, a specific focus should be placed on personnel who perceive no need for education despite having low scores; managers must investigate the underlying reasons for these perceptions to reduce resistance to training. By shifting from a prohibitionist culture to an engaged and positive one, the K‐DP‐SAI can serve as a vital instrument for ensuring that digital technology supports, rather than compromises, the professional integrity of the nursing workforce.

### 4.2. Limitations

There are several limitations to this study as mentioned below.

First, a ceiling effect was observed in several items, particularly those involving ethical violations such as Item 11 (uploading patient images without consent) and Item 25 (exploiting patients for personal or financial gain). Although these items reflect core ethical principles that nurses are expected to uphold, the self‐report format likely triggered social desirability. This would lead to uniformly high scores and limited variability, resulting in lower item discrimination and their eventual exclusion during factor analysis.

Second, direct cross‐cultural comparisons are currently constrained by the lack of international validation studies among nursing populations. While our findings revealed a consolidated structure, it remains to be fully elucidated whether this divergence stems from specific cultural nuances within the nursing profession or the South Korean context itself. Consequently, future research involving rigorous cross‐cultural structural comparisons is essential to clarify the global applicability and robustness of the DP‐SAI.

Third, although ethical safeguards constitute the foundation of digital professionalism, recent literature emphasises the value of proactive features, such as reflective practice and professional digital engagement. Given that the concept is continuously evolving, future research should integrate attitudinal and perceptual measures into the K‐DP‐SAI, which primarily captures behavioural aspects grounded in professional ethical guidelines. This would enable a more balanced and comprehensive assessment of the construct.

## 5. Conclusion

This study validated a digital professionalism measure for nurses in the South Korean context through the translation and cultural adaptation of the DP‐SAI. To our knowledge, this is the first study to translate and validate a digital professionalism instrument for nurses and healthcare providers in South Korea. During this process, the factor structure was reconfigured to reflect nursing professional and linguistic nuances while maintaining the instrument’s original intent. The K‐DP‐SAI demonstrated acceptable convergent and discriminant validity in both EFA and CFA. As the first validated digital professionalism instrument for nurses and healthcare providers in South Korea, this study provides a basis for further research, education and professional development in this emerging field.

## Author Contributions

Suyeon Ban, Ja‐Yeoung Lee and GyeongAe Seomun contributed to the conceptualisation and design of the study. Suyeon Ban and Ja‐Yeoung Lee, acting as coprincipal investigators, undertook literature review, cross‐cultural adaptation process, preliminary testing and validation of the Korean version. Suyeon Ban and Ja‐Yeoung Lee drafted the initial manuscript, while GyeongAe Seomun provided supervision throughout the entire research process and contributed to the revision of the text.

## Funding

No funding was received for this research.

## Disclosure

All the authors have read, reviewed and approved the final version of the manuscript for submission.

## Conflicts of Interest

The authors declare no conflicts of interest.

## Supporting Information

Additional supporting information can be found online in the Supporting Information section.

## Supporting information


**Supporting Information 1** Appendix 1: the STROBE checklist is provided as supporting information to demonstrate adherence to reporting guidelines for observational studies and is referred to in the Methods section.

**Supporting Information 2** Appendix 2: nurses’ perception of social media use contains the survey items used to assess nurses’ perceptions of social media use in professional contexts and is provided as supporting information. This appendix is referred to in the Methods section.

## Data Availability

The data that support the findings of this study are available from the corresponding author upon reasonable request.
