# Supplementary material for: Translation, Cultural Adaptation and Validation of a Korean Version of the Digital Professionalism Self‐Assessment Instrument for Nurses
Source: J Nurs Manag. 2026 Jul 6;2026:7991455. doi: 10.1155/jonm/7991455 (PMC13334282; doi:10.1155/jonm/7991455)
Supplement: Supplementary file 1 — Supporting Information 1 Appendix 1: the STROBE checklist is provided as supporting information to demonstrate adherence to reporting guidelines for observational studies and is referred to in the Methods section. [file JONM-2026-7991455-s002.doc]

STROBE Statement—Checklist of items that should be included in reports of ***cross-sectional studies***

|  | Item No | Recommendation | Answer |
| --- | --- | --- | --- |
| **Title and abstract** | 1 | (*a*) Indicate the study’s design with a commonly used term in the title or the abstract | The title clearly described the study design.  *“Translation, Cultural Adaptation, and Validation of a Korean Version of the Digital Professionalism Self-Assessment Instrument for Nurses”* |
|  |  | (*b*) Provide in the abstract an informative and balanced summary of what was done and what was found | The abstract summarised the aim, methods, key findings, and conclusions in a balanced manner  (page 1) |
| Introduction | | |  |
| Background/rationale | 2 | Explain the scientific background and rationale for the investigation being reported | The scientific background and rationale for translating and validating the K-DP-SAI were described in the introduction.  (page 2-3) |
| Objectives | 3 | State specific objectives, including any prespecified hypotheses | The study objectives were clearly stated in the Introduction. No prespecified hypotheses were specified, as this study aimed to translate and validate a measurement instrument (page 3) |
| Methods | | |  |
| Study design | 4 | Present key elements of study design early in the paper | The key elements of study design were described in the beginning of methods section  *The K-DP-SAI was developed in two distinct phases. Phase 1 utilised the six-step guideline for translation and cross-cultural adaptation from Sousa and Rojjanasrirat [14] and Cruchinho et al. [17] to ensure a rigorous adaptation process and psychometric evaluation (page 3-4)* |
| Setting | 5 | Describe the setting, locations, and relevant dates, including periods of recruitment, exposure, follow-up, and data collection | The study setting, participant recruitment, and data collection period were described in Sections 2.2 and 2.2.1 of the Methods section. (Page 7) |
| Participants | 6 | (*a*) Give the eligibility criteria, and the sources and methods of selection of participants | Inclusion and exclusion criteria were described in Section 2.2.1 of the Methods section (Page 7).  *Participants were required to work in clinical settings and use at least one social media platform (such as Facebook, Instagram, YouTube, KakaoTalk, or blogs) at least once a day, while those not involved in direct patient care were excluded.* |
| Variables | 7 | Clearly define all outcomes, exposures, predictors, potential confounders, and effect modifiers. Give diagnostic criteria, if applicable | N/A  (No exposure, predictor, confounder, or effect modifier variables in this psychometric validation study) |
| Data sources/ measurement | 8* | For each variable of interest, give sources of data and details of methods of assessment (measurement). Describe comparability of assessment methods if there is more than one group | Data were collected using K-DP-SAI, translated and culturally adapted from the original DP-SAI in this study, along with a questionnaire assessing participant characteristics and social media use |
| Bias | 9 | Describe any efforts to address potential sources of bias | Potential bias were addressed through translation and cross-cultural adaptation procedures and random allocation of participants to the EFA and CFA samples. |
| Study size | 10 | Explain how the study size was arrived at | The study size was determined according to recommended sample size criteria for psychometric validation studies and factor analysis. (page 7)  *Based on the recommendation of having at least 10 participants per item, a minimum sample size of 280 was required for each of the exploratory factor analysis (EFA) and confirmatory factor analysis (CFA) [20–22]. To accommodate a 10% dropout rate, a total of 616 participants were recruited.* |
| Quantitative variables | 11 | Explain how quantitative variables were handled in the analyses. If applicable, describe which groupings were chosen and why | Quantitative variables were used descriptive purposes of subjects, as this was a psychometric validation study rather than an analytical observational study. |
| Statistical methods | 12 | (*a*) Describe all statistical methods, including those used to control for confounding | The Methods section describes the statistical procedures for psychometric evaluation, including EFA, CFA, reliability testing, and known-groups validity. No confounding variables were considered, as this was a psychometric validation study. |
|  |  | (*b*) Describe any methods used to examine subgroups and interactions | Known-groups validity was performed by comparing participants according to their perceived need for education regarding social media ethics and professionalism. No interaction analyses were conducted. |
|  |  | (*c*) Explain how missing data were addressed | No missing data were observed |
|  |  | (*d*) If applicable, describe analytical methods taking account of sampling strategy | Not applicable |
|  |  | (*e*) Describe any sensitivity analyses | No sensitive analysis was conducted |
| Results | | |  |
| Participants | 13* | (a) Report numbers of individuals at each stage of study—eg numbers potentially eligible, examined for eligibility, confirmed eligible, included in the study, completing follow-up, and analysed | The numbers of participants allocated to the EFA and CFA were reported in the Results section (page 10) , with a clear description of the study sample in Methods section |
|  |  | (b) Give reasons for non-participation at each stage | No participants were excluded after eligibility assessment |
|  |  | (c) Consider use of a flow diagram | Participant flow was described in the text |
| Descriptive data | 14* | (a) Give characteristics of study participants (eg demographic, clinical, social) and information on exposures and potential confounders | Participant characteristics, including demographic and social media use were reported in Table 1 and appendix 1, separately for the EFA and CFA sample. sNo exposure or confounding variables were considered, as this was a psychometric validation study. |
|  |  | (b) Indicate number of participants with missing data for each variable of interest | No missing data were observed. |
| Outcome data | 15* | Report numbers of outcome events or summary measures | All outcomes derived from the statistical analyses were reported in the Results section separately for the EFA and CFA |
| Main results | 16 | (*a*) Give unadjusted estimates and, if applicable, confounder-adjusted estimates and their precision (eg, 95% confidence interval). Make clear which confounders were adjusted for and why they were included | No adjusted estimates were calculated because this was a psychometric validation study. |
|  |  | (*b*) Report category boundaries when continuous variables were categorized | Continuous variables were not categorised for analytical purposes. |
|  |  | (*c*) If relevant, consider translating estimates of relative risk into absolute risk for a meaningful time period | Relative and absolute risk estimates were not relevant to this psychometric validation study. |
| Other analyses | 17 | Report other analyses done—eg analyses of subgroups and interactions, and sensitivity analyses | No subgroup, interaction, or sensitivity analyses were conducted according to the aim of this study. |
| Discussion | | |  |
| Key results | 18 | Summarise key results with reference to study objectives | The key results were presented and discussed in relation to the study objectives (page 11) |
| Limitations | 19 | Discuss limitations of the study, taking into account sources of potential bias or imprecision. Discuss both direction and magnitude of any potential bias | The limitations of the study were discussed, including potential social desirability bias, limited opportunities for cross-cultural comparison, and the behavioural focus of the instrument (page 15) |
| Interpretation | 20 | Give a cautious overall interpretation of results considering objectives, limitations, multiplicity of analyses, results from similar studies, and other relevant evidence | The Discussion section interpreted the findings in relation to the study objectives, previous research, and the South Korean nursing context, while acknowledging the study limitations. |
| Generalisability | 21 | Discuss the generalisability (external validity) of the study results | The generalisability of the findings was discussed in limitation, including the need for further validation and cross-cultural comparison in different nursing populations and settings. |
| Other information | | |  |
| Funding | 22 | Give the source of funding and the role of the funders for the present study and, if applicable, for the original study on which the present article is based | No funding was received for this study |

*Give information separately for exposed and unexposed groups.
