# Supplementary material for: Translation, Cultural Adaptation and Validation of a Korean Version of the Digital Professionalism Self‐Assessment Instrument for Nurses
Source: J Nurs Manag. 2026 Jul 6;2026:7991455. doi: 10.1155/jonm/7991455 (PMC13334282; doi:10.1155/jonm/7991455)
Supplement: Supplementary file 2 — Supporting Information 2 Appendix 2: nurses’ perception of social media use contains the survey items used to assess nurses’ perceptions of social media use in professional contexts and is provided as supporting information. This appendix is referred to in the Methods section. [file JONM-2026-7991455-s001.docx]

|  |  |  | EFA(N=308) | CFA(N=308) |
| --- | --- | --- | --- | --- |
|  |  |  | n(%)/mean± SD | n(%)/mean± SD |
| Nurses’ social media use | Self-perception | Negative | 7(2.2) | 10(3.2) |
|  |  | Neutral | 126(40.9) | 122(39.6) |
|  |  | Positive | 175(56.8) | 176(57.2) |
|  | Social perception | Negative | 34(11.1) | 45(14.6) |
|  |  | Neutral | 156(50.6) | 131(42.5) |
|  |  | Positive | 118(38.3) | 132(42.8) |
| Appropriateness of social media use as a nursing professional | Self-perception | No | 68(22.1) | 70(22.7) |
|  |  | Moderate | 118(38.3) | 104(33.8) |
|  |  | Yes | 122(39.6) | 134(43.5) |
|  | Social perception | No | 55(17.9) | 62(20.1) |
|  |  | Moderate | 140(45.5) | 119(38.6) |
|  |  | Yes | 113(36.7) | 127(41.2) |
| Need for education on ethics and professionalism related to social media use | | Yes | 262(85.1) | 272(88.3) |
|  |  | NO | 46(14.9) | 36(11.7) |
| Completion of ethics/professionalism education on social media use | Hospital |  | 3.23±0.87 | 1.51±0.90 |
|  |  |  |  |  |
|  | Individual |  | 1.85±1.10 | 1.12±0.32 |
| **Appendix 1. Nurses’ perception of social media use**  Note. N=Total number of sample, n=Portion of total sample, SD=Standard deviation | | | | |
